# Learning From Instructional Videos: Learner Gender Does Matter; Speaker Gender Does Not

**DOI:** 10.3389/fpsyg.2021.655720

**Published:** 2021-05-28

**Authors:** Claudia Schrader, Tina Seufert, Steffi Zander

**Affiliations:** ^1^School of Education, University of Wuppertal, Wuppertal, Germany; ^2^Institute of Psychology, Ulm University, Ulm, Germany; ^3^Department of General Psychology, Magdeburg-Stendal University of Applied Science, Magdeburg, Germany

**Keywords:** instructional videos, speaker gender, learner gender, situational interest, cognitive load

## Abstract

One crucial design characteristic of auditory texts embedded in instructional videos is the speaker gender, which has received some attention from empirical researcher in the recent years. Contrary to the theoretical assumption that similarity between the speaker’s and the learner’s gender might positively affect learning outcomes, the findings have often been mixed, showing null to contrary effects. Notwithstanding the effect on the outcomes, a closer look at how the speaker’s gender and speaker–learner similarities further determine cognitive variables, such as different cognitive load types, is overdue. Moreover, on the part of the learner, the role of situational interest in the learning topic that might be gender related has been neglected so far. Therefore, this study explored the role of speaker and learner gender and their interaction regarding learning outcomes. We broaden our perspective by investigating the effects of gender-related differences concerning situational interest in the topic being taught and by determining different types of cognitive load. In a 2 (female/male speaker) × 2 (female/male learner) within- and between-subject design, 95 students learned about female and male human sexual maturity with an instructional video containing auditory explanations. Analysis results indicate that speaker gender and speaker–learner gender similarity had no impact on learning gains, situational interest, and cognitive load types. However, the results demonstrate that learner’s gender, especially for the topic of female sexual maturity, matters the most in line with the assessed variables. Compared with males, females had higher learning gains, reported higher interest in the topic, and invested more germane cognitive resources. Thus, instructional designers may want to consider learner gender-dependent interest and how it can be triggered when creating videos with auditory explanations.

## Introduction

Learning from instructional videos is an effective means of knowledge acquisition and understanding, which is increasingly used in educational learning settings. A large amount of empirical work on learning with videos has investigated a range of cognitive principles of learning and instructional design (for an overview, see e.g., the special issue of *Computers in Human Behavior* edited by de Koning et al., 2018) based on the cognitive theory of multimedia learning (CTML; Mayer, 2005a) and the cognitive–affective theory of learning with media (CATLM; Moreno and Mayer, 2007). For example, supplementing visual presentations, mostly in the form of pictorial presentations, with auditory explanations have been shown to lead to optimal use of learner cognitive resources and, therefore, enhance the learning outcome (e.g., Mayer and Moreno, 2003; for an overview, see Mayer, 2005b). Inserting pauses in instructional videos has been further shown to support learners in structuring, organizing, and integrating the information learned from instructional videos into existing knowledge structures (e.g., van der Meij and van der Meij, 2013; Merkt et al., 2018). Another well-known and essential characteristic of instructional videos is the design of the instructor or speaker. For example, its presence or absence (e.g., van Wermeskerken et al., 2018) and/or the position of the instructor shown in instructional videos (e.g., Boucheix et al., 2018) has been analyzed on a cognitive perspective. The present study aims to dig deeper into the role of the voice of the speaker by addressing the speaker gender that might influence the effectiveness of instructional videos. The voice of the speaker is an important design feature, which is easy to manipulate. What is more, instructional videos are very often produced without a visible speaker. Particularly, nowadays, where instructional videos needed to be produced without technical effort and lengthy production steps, adding narratives to visuals is a very common format of instructional videos. Regarding an effective design of such a format, the questions arise whether learning is more effective when a speaker’s voice is either female or male and whether a possible effect also depends on the interplay with learner gender. Whereas it has been theoretically argued that there are positive effects on cognitive processes and outcomes when learning from a speaker with the same gender as the learner, existing empirical research, however, reveals inconsistent to null findings (e.g., Arroyo et al., 2009; Rodicio, 2012; Hoogerheide et al., 2016, Hoogerheide et al., 2017, 2018). However, even if there are no consistent results for learning outcomes, it seems plausible that learners might be affected motivationally by the gender of the speaker’s voice in interaction with their own gender. In fact, there are indications in research of a possible impact of motivational learner attributes such as learners’ situational interest about the provided learning topic. Thus, it seems worthwhile to add this motivational perspective of gender effects as it has been rarely considered in research on instructional videos so far.

Considering the aforementioned limitations, this study was conducted to expand the existing research on speaker gender in instructional videos by examining the effect of speaker gender and the interaction between speaker and learner gender from both cognitive and motivational perspective. More specifically, we will analyze the effects of speaker gender and its similarity with learner gender on learning outcomes when learning about female and male human sexual maturity with an instructional video. As specific prerequisite of the study, a separate look will be done not only on specific learning outcomes representing either the learning content of the female or of the male sexual maturity. Furthermore, different types of cognitive load will be analyzed underlying the cognitive perspective. From a motivational perspective, we will include the motivational variable of learners’ situational interest in human sexual maturity. Whether there are gender-related differences in situational interest and the extent to which gender-dependent situational interest enhances learning-related cognitive processes and outcomes are investigated.

The study is expected to have important implications for tailoring a speaker voice to learners in a manner that would improve their performance when learning with instructional videos.

## Effects of Speaker Gender When Learning From Instructional Videos

Studies on the so-called voice principle demonstrate that learners prefer learning from a human voice over machine-synthesized voices, which leads to more mental effort investment and to higher learning success (e.g., Atkinson et al., 2005). This has been explained by the theoretical assumptions outlined in the social agency theory (Bandura, 1989) and media equation theory (Reeves and Nass, 1996), arguing that social cues, such as a human voice, lead to the perception of being in some kind of social communication (Linek et al., 2010).

Despite investigating whether human voices generally affect learning in a multimedia setting, a few studies (e.g., Arroyo et al., 2009; Linek et al., 2010; Rodicio, 2012; Hoogerheide et al., 2016, 2018) have examined whether one can learn more effectively from either a female or a male instructor or speaker. This question is highly relevant as gender is argued to be also one of the first and strongest social cues that others notice in a learning environment (Contreras et al., 2013). Empirical studies in which only the instructor’s voice can be heard partly demonstrate that the learning impact of a male or female speaker depends on perceived attributes such as the speakers’ attractiveness or gender stereotypes such as the assumed appropriateness of a speaker concerning the learning topic. For example, the finding of the study of Linek et al. (2010) revealed that, due to a higher perception of a female speakers’ voice attractiveness, learners invested higher mental effort in terms of the overall number of cognitive resources when learning mathematics with auditory explanations and reached better learning outcomes. In contrast, in the study of Rodicio (2012), learners perceived male speakers as more competent in contrast to female speakers when learning about more typically male-based topics such as geology or mathematics, which resulted in higher learning outcomes. A similar result has been found in the study of Hoogerheide et al. (2018) where the instructors could not only be heard but also seen. They found that male models demonstrating troubleshooting electrical circuits were perceived as more competent concerning the learning topic, although the models’ gender did not have an impact on the invested mental effort nor on the learning outcomes.

In addition, the interaction between speaker and learner gender has also been analyzed in research on instructional videos. There exists a widespread assumption in the literature that a similarity between speaker and learner gender fosters motivation, affect, and cognitive processes for learning. The underlying mechanism is the process of social comparison, according to the so-called model-observer similarity hypothesis (Schunk, 1991; Bandura, 1994) and the similarity-attraction hypothesis (Moreno and Flowerday, 2006). Theoretically, it is assumed that the learner may be more willing to pay attention to a much similar person and may use similarity as an indicator of his or her own success (Bandura, 1971). The learner will, due to the perceived similarity, also feel to be more confident about succeeding and may adjust and, hence, decrease mental effort (Linek et al., 2010). Indeed, various studies underline the positive effects of gender similarity between speaker and learner on motivational and affective variables. More specifically, when given the choice, learners, in general, prefer someone of the same gender (e.g., Weeks et al., 2005; Linek et al., 2010; Ozogul et al., 2013), which leads to a more positive evaluation of, and more trust in, the speaker (Lee et al., 2007). Furthermore, the results of the study by Weeks et al. (2005) show a development in self-efficacy of about 70-year-old learners when instructed for physical exercise via a video instructor. However, when it comes to positive effects of gender similarity on cognitive processes and outcomes, the results of existing empirical studies are less convincing. No interaction effect of speaker and learner gender on mental effort (e.g., Linek et al., 2010) and on learning outcomes (e.g., Hoogerheide et al., 2016, Hoogerheide et al., 2017, 2018) was found when investigating video instructions on natural science topics.

Although one has to keep in mind differences in the mentioned empirical studies (e.g., type and visibility of instructor or speaker, type and age of students, and learning topic) that could have led to the inconsistent findings, we argue for expanding the research in this field in order to shed a different and broader perspective.

First, until now, the impact on cognitive processes has only been investigated by Linek et al. (2010) and Hoogerheide et al. (2018). Hoogerheide et al. (2018) only investigated cognitive load in terms of the perceived investment of mental effort, which was not affected by either the speaker’s or learner’s gender. Linek et al. (2010) additionally differentiated learners perceived cognitive load in terms of intrinsic, extraneous, and germane load with the NASA-TLX (Hart and Staveland, 1988). They found increased effort ratings after listening to the female voice, but no effects on the differentiated scales of cognitive load. However, as the NASA-TLX scales cannot be clearly aligned to the three types of cognitive load as intended by cognitive load theory, we aim at using a validated differentiated measure to shed light on the actual cognitive processes while learning (Klepsch et al., 2017). From a cognitive perspective, the learning and instructional design can have an impact on learner cognitive load—the load imposed during learning as a result of having to process information for a particular task within limited-capacity working memory (Paas and Sweller, 2014). As proposed in cognitive load theory (CLT) (Sweller, 2011), two types of cognitive load are assumed—extraneous and intrinsic cognitive load. Whereas extraneous cognitive load results from pitfalls in the instructional design, intrinsic cognitive load is argued to be caused by the intrinsic complexity of learning information or tasks used to achieve specific learning goals in interaction with learner expertise. In addition to both cognitive load types that determine the required working memory resources, the concept of actually allocated working memory resources is also used in the literature as third cognitive load type. It was referred to as germane cognitive load in former conceptualizations of CLT (Sweller et al., 1998) and is now specified as germane cognitive resources, i.e., those working memory resources that are actually allocated to deal with information intrinsic to learning goals (Kalyuga, 2011). These germane resources depend on a student’s level of engagement and motivation during learning. This is the reason why we investigated all the three types of load, as particularly these actively invested resources could be affected by social cues and the effects of gender similarity. Thus, with this differentiated measurement approach, we strive at specifying which aspects of the design affect which aspect of the cognitive affordances in terms of cognitive load (Klepsch and Seufert, 2020).

Second, as findings of the aforementioned studies do not fully confirm the importance of considering the speaker gender when designing auditory explanations in instructional videos, we argue that these differences might depend on learner gender and related situational interest in the topic.

## Learners’ Situational Interest in the Learning Topic

Interest is characterized as a person–object relationship that leads to engagement or re-engagement. It is differentiated into individual interest (i.e., a trait) and situational interest (i.e., a state) (Hidi and Renninger, 2006). Situational interest, as a focus of this study, emerges and changes in a specific learning situation. It is determined by the characteristics of the specific learning situation such as the individual perceived interestingness of the learning topic or the design of the learning environment (Lewalter and Geyer, 2009). Situational interest can be differentiated into two forms: triggered situational interest and maintained situational interest (e.g., Hidi and Renninger, 2006). Whereas the former is accompanied by focused attention and positive emotions at the beginning, the latter is characterized by a growing sense of value and engagement with an epistemic orientation toward the learning object (Knogler et al., 2015). Both forms of situational interest can help learners to focus on learning and could have a positive influence on the quality of learning behavior, on cognitive activation like using deep learning strategies. As a result, such a focused and deepened learning process could help to improve learning outcome (e.g., Hidi, 1990; Hidi et al., 1992; Rotgans and Schmidt, 2011). Thus, the consideration of situational interest seems to be relatively important.

However, currently, no specific studies exist that investigate whether and how the instructor or speaker gender in line with the provided learning topic as environmental factors affect learners’ situational interest. Not only external, environmental factors can trigger situational interest but also the learner gender as an internal factor might trigger situational interest. Albeit outside research on instructional videos, a large body of research has investigated learners’ interest in topics within the field of science education. The perceived interest in learning mathematics and physics is argued to be higher for males than for females (Häussler and Hoffmann, 2002; Forgasz et al., 2004; Uitto et al., 2006), which might explain the inconsistent findings on learning outcomes in the studies on the speaker–learner gender interplay when learning with instructional videos mentioned above.

With human biology as the learning topic in the instructional video in our study, female learners were shown to be more interested in biology (e.g., Dawson, 2000; Cakmakci et al., 2012; Hagay et al., 2013), specifically in human biology, reproduction, and sexuality. In contrast, male learners prefer ecological and cellular phenomena (e.g., Dawson, 2000; Jenkins and Pell, 2006; Uitto et al., 2006; Hagay et al., 2013).

In conclusion, differences in situational interest in the topic exist between female and male learners. However, until now, the role of situational interest in a specific topic has rarely been explored in the literature on auditory explanations in instructional videos. Other studies investigated learners’ self-efficacy as a motivational variable, which could be affected by the speaker’s voice (e.g., Weeks et al., 2005; Hoogerheide et al., 2016). However, the self-efficacy ratings mostly referred to the belief of being able to solve a problem or perform an exercise. With more abstract and knowledge-based topics, a learner’s situational interest should be a crucial indicator of motivational processes.

## Present Study

For the present study, an instructional video for learning about female and male human sexual maturity was developed and used to investigate the role of speaker and learner gender and their interplay in terms of learning gains. In addition, the study explored their effect on the amount of intrinsic, extraneous, and germane cognitive load. For this purpose, we used the differentiated measure of Klepsch et al. (2017) to distinguish between the different load types. Besides, learners’ situational interest in human sexual maturity and within differences in situational interest between females and males was assessed. Both the measurement of cognitive load and situational interest might contribute to an understanding of the impact of cognitive and motivational factors while learning.

Concerning the impact of *speaker gender*, it is often argued that when either a female or a male speaker is perceived as competent regarding a learning topic, learners will have higher motivation. They will invest more learning-related cognitive resources, which, in turn, should improve learning outcomes (Linek et al., 2010). The topic used in this study, i.e., female and male human sexual maturity, is proven a typical female topic (e.g., Dawson, 2000; Cakmakci et al., 2012; Hagay et al., 2013). Thus, one might conclude that in contrast to a male speaker, a female speaker will be perceived as more competent in the topic. This, in turn, is argued to lead to higher learning in contrast to a male speaker. However, a few recent studies (Hoogerheide et al., 2016) have reported no significant differences between either a female or a male voice in terms of learning outcomes. Based on these findings, we assume no main effect of speaker gender on learning outcomes.

For cognitive load measurement, as an expansion of existing research, we expect no effects of speaker gender on intrinsic load either because the learning content and its inherent complexity do not change depending on the speakers’ voice. Information processing should also be equally straightforward when listening to a female or a male voice, as we are familiar with voices of every gender in everyday life, as well as in learning settings. Thus, it seems implausible that extraneous load should be affected by a speaker gender as well. For germane load, there could be possible effects of speaker gender, i.e., a higher investment of germane resources when learning with a female speaker, on the assumption that she is perceived to be more competent with regard to the topic of human sexual maturity. Germane cognitive load might be also triggered through altered motivational variables (e.g., Weeks et al., 2005).

Regarding the effects of *learner gender*, we assume differences of male and female learners in learning process variables such as situational interest and germane cognitive load. On the learners’ side, there appears to be a gender specificity of topics, where either male or female learners are superior. Learners’ situational interest in the learning topic could explain this specificity. Although previously not investigated in the context of gender, in line with auditory explanations, a significant amount of evidence has been presented to indicate that situational interest in human biology and sexuality is higher among female learners compared with males (e.g., Dawson, 2000; Uitto et al., 2006). Thus, as the learning content of the instructional video used in our study is about the sexual maturity of both female and male humans, we assume the existence of a main effect of learner gender on situational interest, i.e., a higher interest among females compared with males. Coming along with an increased situational interest, learners might also invest more germane cognitive resources. We, hence, expect a main effect of learner gender for germane cognitive load with higher scores for female compared with male learners, whereas there should be no differences in intrinsic and extraneous cognitive load between females and males. Accordingly, the outcome of such higher situational interest and germane cognitive load is a deeper learning that should be accompanied with a higher learning gain. Thus, we also assume a main effect of learner gender on learning gains, with higher learning gain for female learners, compared with male learners, independent of speaker–learner similarity.

According to the *interaction of speaker and learner gender*, the findings of the majority of the aforementioned recent empirical studies do not support the similarity-attraction hypothesis. Thus, for our study, we do not expect interaction effects between speaker and learner gender for all dependent measures.

## Materials and Methods

### Participants and Experimental Design

The participants comprised 95 students (48 females, 47 males, *M*_*age*_ = 23.47, *SD* = 3.67) majoring in psychology, computer science, economics, and engineering science at a German university. The native language for most of the participants (98%) was German, whereas 2% of the participants stated they had spoken German for more than 20 years.

The experiment involved a 2 × 2 experimental design consisting of four experimental groups with the speaker gender (female vs. male speaker) in the auditory explanations given in the instructional video and learner gender (female vs. male) as an independent between-subject factor. The participants were quasi-randomly assigned (i.e., matched according to gender; for instance, for each female learner allocated to the video with a female speaker, another female learner was allocated to the video with a male speaker) either to the video with a female speaker (22 males, 26 females) or a male speaker (24 males, 24 females). In order to investigate the formulated assumptions stated above, the resulting learning gains in total and separately for gender-specific topics of either female or male sexual maturity, was the dependent measure. As additional dependent variables, we analyzed students’ situational interest as well as their perceived cognitive load, differentiated in terms of intrinsic, extraneous, and germane cognitive load.

### Learning Material

**FIGURE 1:**
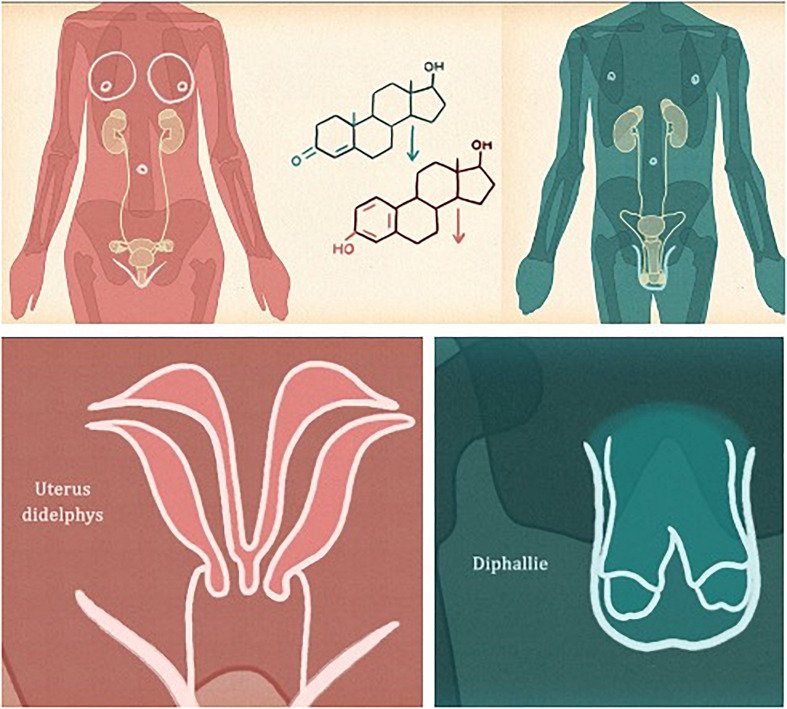
Screenshots of the video-based learning environment.

For the study, an instructional video was developed to teach students about the sexual maturity of both female and male humans (see Figure 1). The instructional video consists of visual animations with static pictures about sexual maturity. More specific, it consists of three constitutive parts, i.e., reproduction, anatomic dysplasia, and the development of hormone levels during the lifespan for both female and male humans. The information presented in the video was given through auditory explanations, i.e., it was supplemented by a speaker’s voice explaining the information presented in the video. The auditory text length is 457 words. Additionally, a few keywords or numbers were displayed in printed form on the slides. The time of the video was approximately 4 min.

In order to investigate the impact of the speakers’ gender, two versions of the video were created. Although, in both video versions, the same explanations were made in the same number of words and amount of time for all three constitutive parts, the versions differed in terms of the speaker gender, i.e., one with a female speaker and one with a male speaker assuming the role of the instructor. Professional speakers recorded both versions.

### Measures

#### Situational Interest

Learners’ situational interest in the sexual maturity and reproduction of male and female humans was assessed after learning. For this purpose, the short version of the situational interest questionnaire created by Lewalter and Geyer (2009) was adapted. The survey consisted of six items, which assessed the triggered interest (e.g., “I was fully focused on the topic about human sexual maturity”) and the maintained interest (e.g., “I would like to receive further information on sexual maturity”). Each of them was assessed with three items using a seven-point Likert scale anchored from 1 (very much disagree) to 7 (very much agree) (Cronbach’s α = 0.86). As these two facets of situational interest were highly correlated (*r* = 0.64), only one score for situational interest was calculated (Cronbach’s α = 0.86). This is in line with the finding of Specht (2013) using the short version of the situational interest questionnaire.

#### Cognitive Load

Learners were asked about their cognitive load after learning with use of the video by using the cognitive load questionnaire created by Klepsch et al. (2017). The questionnaire was validated in follow-up studies (e.g., Klepsch and Seufert, 2020). It includes two items on intrinsic cognitive load (e.g., “For this task, many things need to be kept in mind simultaneously”), three items on extraneous cognitive load (e.g., “During this task, it was exhausting to find the important information”), and two items on germane cognitive load (e.g., “My point while dealing with the task was to understand everything correctly”). All items were measured on a seven-point rating scale ranging from 1 (very low) to 7 (very high). The reliability, as measured by Cronbach’s α, was 0.84 for intrinsic cognitive load, 0.70 for extraneous cognitive load, and 0.77 for germane cognitive load.

#### Learning Gain

In order to access learner outcomes, their learning gain was measured by computing the differences between the posttest score (Cronbach’s α = 0.55) and the pretest score (Cronbach’s α = 0.58). Three questions in each of the tests had to be excluded due to a difficulty index lower than 0.20 or higher than 0.80. To this end, both tests comprised 12 questions on human sexual maturity including six questions about females’ sexual maturity and six questions about males’ sexual maturity. All questions assessed retention knowledge facets (e.g., “Please name the hormone with the following structure…”). The two test versions of the pre- and posttests were parallel to each other, that is, the content of the questions was equivalent across both tests, but they differed in the sequence of presentation and structure. A maximum of 12 points could be earned on both tests in total. Partially correct answers were given partial credits. When learners, for example, listed two out of four terms required, they were granted with 0.5 points, and for only one term, they were granted 0.25 points. For the gender-specific test parts, a maximum of six points could be achieved.

### Procedure

For the experiment, participants were individually tested in a laboratory setting for about 30 min. Participants were first provided with a demographic questionnaire (e.g., on age, gender, and educational level), followed by the pretest according to participants’ prior knowledge of sexual maturity. Participants were given approximately 10 min to complete this first phase of the procedure. Afterward, participants received brief instructions about the video before starting to learn with it. All participants viewed and heard the same video; the only manipulation of this experiment was the gender of the speaker. All groups tended to work through the whole video within a period of 4 min. Afterward, the participants completed the cognitive load rating scale and the situational interest rating scale. Finally, these scales were followed by the posttest on learning outcome with regard to the provided content.

## Results

The data were analyzed by means of a 2 × 2 analysis of variance (ANOVA) concerning the speaker gender (female vs. male speaker) and learner gender (female vs. male) for learners’ situational interest and cognitive load. The 2 × 2 analysis of variance (ANOVA) was further used to analyze the learning gain per learner, which, in a first step, was computed by subtracting the score on the knowledge posttest by the score on the pretest. To ensure that female and male learners had comparable prior knowledge on the topic, differences in the pretest were tested using the 2 × 2 analysis of variance (ANOVA) again. There was no significant difference in the overall prior knowledge between all females and males who participated in this study (*p* = 0.66), no difference in prior knowledge of them between the different video conditions (*p* = 0.42), as well as no significant interaction of both (*p* = 0.92). Hence, no initial differences needed to be corrected for the analyses. Descriptive statistics of each of the measured variables can be found in Table 1.

**TABLE 1:** Mean scores and standard deviation for each variable.

	**M (SD)**			
	**Female speaker**	**Male speaker**	**Female speaker**	**Male speaker**
		
	**Female learner**		**Male learner**	
Prior knowledge total	4.46 (1.21)	4.22 (1.47)	4.32 (1.08)	4.12 (1.45)
Learning gain total	4.38 (1.85)	4.74 (2.35)	3.27 (1.79)	3.09 (1.45)
Learning gain FSM	2.62 (1.44)	2.91 (1.31)	1.91 (1.15)	1.67 (0.91)
Learning gain MSM	1.76 (1.30)	1.83 (1.49)	1.36 (1.13)	1.42 (1.49)
Intrinsic cognitive load	4.29 (1.49)	3.27 (1.68)	2.79 (1.57)	2.95 (1.37)
Extraneous cognitive load	2.16 (1.07)	2.32 (0.71)	2.59 (1.38)	2.56 (1.11)
Germane cognitive load	5.37 (1.32)	5.48 (1.29)	4.75 (1.58)	4.58 (1.34)
Situational interest	4.91 (1.11)	4.84 (1.07)	4.34 (1.17)	4.20 (1.15)

*FSM, female sexual maturity; MSM, male sexual maturity.*

### Effects of Speaker Gender

Investigating the main effect of the speaker gender on learners’ situational interest revealed no significant difference (*p* = 0.65).

Furthermore, analyses of cognitive load revealed no significant main effect of the speaker gender for intrinsic (*p* = 0.18), extraneous (*p* = 0.77), as well as germane cognitive load (*p* = 0.93).

Most importantly, the results concerning whether the speaker gender impacts learners’ overall learning gain also showed no significant findings (*p* = 0.79). Moreover, with regard to the sexual maturity of female *or* of male humans, the speaker gender did not account for learning about the *sexual maturity of females* (*p* = 0.91) and for learning about the *sexual maturity of males* (*p* = 0.83).

### Effects of Learner Gender

The 2 × 2 ANOVA, with situational interest as a between factor, revealed a significant main effect of learner gender [*F*(1,91) = 6.73, *p* = 0.04, η^2^ = 0.07]. The descriptive statistics in Table 1 show that female learners developed a higher situational interest in the topic of sexual maturity compared with males.

Besides, for intrinsic cognitive load, a significant main effect of learner gender [*F*(1,91) = 7.98, *p* < 0.01, η^2^ = 0.08] was demonstrated. As shown in Table 1, females reported a higher intrinsic load compared with males. Although no significant effect for extraneous cognitive load between female and male learners was found (*p* = 0.14), the results for germane cognitive load also showed a significant main effect [*F*(1,91) = 7.01, *p* = 0.04, η^2^ = 0.07). As for the intrinsic cognitive load, females reported a higher germane cognitive load in contrast to males.

Analyses of the learning gains show a significant main effect of learner gender on the total learning gain [*F*(1,91) = 13.13, *p* = 0.001, η^2^ = 0.12). As shown in the descriptive statistics in Table 1, females performed significantly better than males. Furthermore, the main effect of learner gender concerning a learning gain on the separate topic of *sexual maturity of females* was also significant [*F*(1,91) = 14.96, *p* = 0.001, η^2^ = 0.14), with better scores for female learners. For performance in relation to a gain in knowledge about the *sexual maturity of males*, however, no main effect of learner gender (*p* = 0.12) was revealed.

### Interactions Between Speaker and Learner Gender

For situational interest, the interaction between the speaker and learner gender was not significant (*p* = 0.86). Besides, we did not find any interaction effect between the speaker and learner gender for the three types of cognitive load (*p* = 0.06 for intrinsic cognitive load, *p* = 0.70 for extraneous cognitive load, and *p* = 0.63 for germane cognitive load).

Finally, no interaction effects concerning total learning gains for the speaker × learner gender were found (*p* = 0.46). As for the total learning gain, no other interaction effects were found for both the knowledge gain in *sexual maturity of females* (*p* = 0.29) and for the knowledge gain in *sexual maturity of males* (*p* = 0.99).^1^

## Discussion

The aim of the present study was to analyze how the design of a speaker’s voice in instructional videos could affect learning. Compared with video production with fully visible models, narrations in instructional videos can be easily produced and altered. Thus, the question on how to design narration is of practical relevance. However, from a sociocognitive perspective, the speakers’ gender is one of the crucial characteristics of auditory texts used in instructional videos that could influence learning. Furthermore, we did not only focus on the effects of speaker gender but also on the similarity of speaker and learner gender or learner gender alone. For this reason, we not only addressed the cognitive perspective by focusing on learning outcomes and perceived cognitive load but also incorporated possible effects on motivational aspects that are important for learning. Concerning the latter, we explored the role of learner gender- and instruction-dependent situational interest in the topic of sexual maturity, which should be affected by the learners’ relation to the topic but also could be determined by situational factors such as the speaker gender.

One of the questions we addressed with respect to the instructional design of the video was whether speaker gender, i.e., learning with either a female or a male voice, would affect situational interest, types of cognitive load, and learners’ learning gain. Here, we found no effects. Learners’ situational interest was not affected by the speaker’s gender. In addition, for learning gains, as well as learners perceived cognitive load with all the subfacets of intrinsic, extraneous, and germane cognitive load, we did not find any differences in learning with either a female or a male speaker. This is in line with previous findings in the literature (e.g., Linek et al., 2010).

Concerning the question about whether a similarity or non-similarity of speaker and learners’ gender could affect learning, we argued that similarity should not affect learning outcomes or intrinsic and extraneous cognitive load. In line with previous studies (Linek et al., 2010; Hoogerheide et al., 2016, Hoogerheide et al., 2017, 2018), which also failed to prove the model-observer similarity hypothesis and the similarity-attraction hypothesis, we found no interaction in the case of learning outcomes, in general, nor for any of the subtopics. Furthermore, as assumed, no effects of intrinsic or extraneous load were found. While other studies (e.g., Weeks et al., 2005) confirmed the effects of similarity on motivational variables, such as self-efficacy, we explored the effects of the similarity of the speaker’s gender on situational interest, hoping to find similar effects. However, this turned out not to be the case. The similarity did not seem to alter the appraisal of the situation to any noteworthy extent. Consequently, we did not find an effect on learners invested germane load. The question arises as to why situational interest is not affected in the same way as self-efficacy. One possible explanation is that self-efficacy highly depends on learners’ content-related self-concept. This might be more easily affected by the perceived competence of the instructor, which can be used as a cue for one’s own feelings of competence and self-efficacy. Gender similarity might therefore help to foster learners’ self-efficacy (Weeks et al., 2005). In contrast, situational interest is less strongly related to learners’ self-concept and, thus, less affected by the perceived competence of the instructor. To further investigate the interplay between learners’ interest in the topic and their topic-related self-concept, it again would be interesting to analyze the perceived competence of themselves in terms of self-efficacy and that of the instructor.

Finally, previous studies as well as the findings of the present study demonstrate the significance of learner gender when learning with videos. We found evidence that the situational interest in the topic is a gender-related factor. Compared with males, females showed a higher interest in human sexual maturity than males, invested a higher germane load, and, in the end, experienced higher learning gains concerning overall knowledge of human sexuality, along with the separate topic of female sexuality. Interestingly, female learners also rated the intrinsic load to be higher than for male learners. The germane load ratings and the increased learning outcomes suggest that they have more intensively dealt with the learning material and, thus, might have “detected” more details and relations within the learning material, resulting in higher ratings of complexity. Overall, we could show that situational interest is, in fact, a sensitive indicator for increased motivation with all the positive effects on learning. As we found a strong trigger for at least a female’s situational interest, in future studies, one should either use a more neutral topic—at the risk of triggering no situational interest for neither gender—or two separate learning issues with one each of interest for either men or women in a cross-over design.

As the difference in learning gains specifically occurred for the topic of female sexuality but not for the separate topic of male sexuality, one could theoretically assume that the interest in sexual maturity linked to the own gender might have contributed to this result. While we assessed learning gains for the separate topics of female and male sexuality, however, we did not measure situational interest and cognitive load in the same separate way. This is another limitation of our study with regard to the effect of speaker and also learner gender. Furthermore, the order of how the topic was provided was not variegated in our study, i.e., the video started with female sexual maturity followed by male sexual maturity. To prevent attention effects due to fatigue failure, this should be considered in further studies.

To sum up, the findings of the reported study did not account for the consideration of speaker gender or the similarity between speaker and learner gender when designing auditory explanations in videos for learning. In contrast, the findings were driven by the gender of learners and by the gender-dependent motivational factor of situational interest in the topic. Therefore, a prevalent purely cognitive perspective should be augmented by a motivational perspective. Further studies should investigate gender-related situational interest in the topic more deeply by comparing strong female-related topics and male-related topics. Only in this way will it be possible to derive practical design recommendations for auditory explanations in instructional videos.

## Data Availability Statement

The raw data supporting the conclusions of this article will be made available by the authors, without undue reservation.

## Ethics Statement

Ethical review and approval was not required for the study on human participants in accordance with the local legislation and institutional requirements. The patients/participants provided their written informed consent to participate in this study.

## Author Contributions

SZ organized, developed, and designed the learning material. CS organized the database and performed the statistical analysis. All authors contributed to conception and design of the study, wrote sections of the manuscript, and contributed to manuscript revision, read, and approved the submitted version.

## Conflict of Interest

The authors declare that the research was conducted in the absence of any commercial or financial relationships that could be construed as a potential conflict of interest.
